# Cost-effectiveness of screening with transcriptional signatures for incipient TB among U.S. migrants

**DOI:** 10.1371/journal.pmed.1004603

**Published:** 2025-05-08

**Authors:** Yuli Lily Hsieh, C. Robert Horsburgh Jr, Ted Cohen, Jeffrey W. Miller, Joshua A. Salomon, Nicolas A. Menzies

**Affiliations:** 1 Interfaculty Initiatives in Health Policy, Harvard University, Cambridge, Massachusetts, United States of America; 2 Harvard Center for Health Decision Science, Boston, Massachusetts, United States of America; 3 Departments of Global Health, Epidemiology, Biostatistics, and Medicine, Boston University, Boston, Massachusetts, United States of America; 4 Department of Epidemiology of Microbial Diseases, Yale School of Public Health, New Haven, Connecticut, United States of America; 5 Department of Biostatistics, Harvard T.H. Chan School of Public Health, Boston, Massachusetts, United States of America; 6 Department of Health Policy, Stanford University School of Medicine, Stanford, California, United States of America; 7 Department of Global Health and Population, Harvard T.H. Chan School of Public Health, Boston, Massachusetts, United States of America; UniversitatsKlinikum Heidelberg, GERMANY

## Abstract

**Background:**

Host-response-based transcriptional signatures (HrTS) have been developed to identify “incipient tuberculosis (TB)”. No study has reported the cost-effectiveness of HrTS for post-arrival migrant screening programs in low-incidence countries. The aim of this study was to assess the potential health impact and cost-effectiveness of HrTS for post-arrival TB infection screening among new migrants in the United States.

**Methods and findings:**

We used a discrete-event simulation model to compare four strategies: (1) no screening for TB infection or incipient TB; (2) ‘IGRA-only’, screen all with interferon-gamma release assay (IGRA), provide TB preventive treatment for IGRA-positives; (3) ‘IGRA-HrTS’, screen all with IGRA followed by HrTS for IGRA-positives, provide incipient TB treatment for individuals testing positive with both tests; and (4) ‘HrTS-only’, screen all with HrTS, provide incipient TB treatment for HrTS-positives. We assessed outcomes over the lifetime of migrants entering the United Stataes (U.S.) in 2019, assuming HrTS met WHO Target Product Profile (TPP) optimal criteria. We conducted sensitivity analyses to evaluate the robustness of results. Our findings show that at a willingness-to-pay threshold of $150,000 per quality-adjusted life-year (QALY) gained, the IGRA-only strategy was the optimal strategy under both healthcare sector and societal perspectives, with an incremental cost-effectiveness ratio (ICER) of $104,138 and $143,103 per QALY gained, respectively. At a willingness-to-pay of $100,000 per QALY gained the IGRA-HrTS strategy appeared optimal. When the cohort was stratified by TB incidence in the country-of-origin, the IGRA-only strategy was optimal for country-of-origin incidence ≥100 per 100,000, and the no-screening strategy was optimal for country-of-origin incidence <10 per 100,000. The IGRA-HrTS strategy was potentially cost-effective with country-of-origin incidence of 10–100 per 100,000, though this result had substantial uncertainty. Results were sensitive to time trends in TB progression risk after U.S. entry.

**Conclusions:**

An HrTS test meeting WHO TPP optimal criteria would be potentially cost-effective for post-arrival screening among a subset of U.S. migrants, but this result was sensitive to multiple factors.

## Author summary

**Why was this study done?**

- In the United States, 71% of reported tuberculosis (TB) cases between 2017 and 2021 occurred in non-US-born persons, and most were attributable to infection acquired before entry.
- The current recommended screening tools (interferon-gamma release assays, IGRA) are useful in identifying TB infection, but many who tested positive with IGRA would not develop TB disease in the future.
- Host-response-based transcriptional signatures (HrTS) are emerging TB screening tools that can identify individuals at higher risk of developing TB disease (incipient TB).
- The goal of this study was to assess the potential health impact and cost-effectiveness of HrTS for post-arrival TB infection screening among new migrants in the United States.

**What did the researchers do and find?**

• We conducted a model-based cost-effectiveness analysis to compare four post-arrival TB screening strategies: (I) no screening for TB infection or incipient TB (‘no-screening’); (II) screen all with IGRA, provide TB preventive treatment for individuals testing positive (‘IGRA-only’); (III) screen all with IGRA followed by HrTS for IGRA-positive persons, provide incipient TB treatment for individuals testing positive with both tests (‘IGRA-HrTS’); and (IV) screen all with HrTS, provide incipient TB treatment for individuals testing positive (‘HrTS-only’).
- We found that the IGRA-only strategy was expected to result in greater reduction in TB cases over lifetime and greater gain in quality-adjusted life years (QALYs), compared to strategies that incorporated HrTS.
- When compared to the no-screening strategy, the IGRA-only strategy resulted in an ICER of $104,138 per QALY gained in the healthcare sector perspective and an ICER of $143,103 in the societal perspective, making it the preferred strategy at a willingness-to-pay threshold of $150,000 per QALY gained. The IGRA-HrTS strategy appeared optimal at a willingness-to-pay of $100,000 per QALY gained.
- When examining across migrants from countries of different TB risks, the IGRA-only strategy was optimal for higher risk groups and the no-screening strategy was optimal for lower risk groups at a willingness-to-pay thresholds of $150,000 and $100,000 per QALY gained.

**What do these findings mean?**

- Our study suggests that HrTS is unlikely to be cost-effective compared to the conventional IGRA test as a post-arrival screening tool among high-risk migrants in the United States.

## Introduction

Without preventive treatment, approximately 5%–10% of healthy individuals infected with *Mycobacterium tuberculosis (Mtb)* will progress to tuberculosis (TB) disease during their lifetime [1,2]. Current WHO guidelines recommend targeting TB preventive treatment to infected persons at highest risk of disease progression. However, current tests for *Mtb* infection, including the interferon-gamma release assay (IGRA) and tuberculin skin test, have low positive predictive values (PPVs) for the proximal onset of TB disease, with only 1%–6% of individuals identified with these tests developing TB within two years [3–6]. The lack of tools for predicting TB disease limits the ability of TB programs to target preventive treatment to those at the highest risk.

In recent years, host-response-based transcriptional signatures (henceforth, HrTS) have been investigated for their potential to identify “incipient TB” based on the association between changes in host transcriptome and risk of disease progression [7,8]. Incipient TB has been defined as “*Mtb* infection that is likely to progress to TB disease in the absence of further intervention but has not yet induced clinical symptoms, radiographic abnormalities, or microbiologic evidence consistent with TB disease” [9]. If HrTS can distinguish incipient TB from non-progressing *Mtb* infection, these tests could facilitate more targeted delivery of interventions to prevent disease progression. WHO has established a Target Product Profile (TPP) for this class of tests, recommending sensitivity and specificity of at least 75% (minimum criteria), and ideally 90% (optimal criteria), for distinguishing individuals who would progress to TB disease within two years [7].

One potential use case of HrTS is for targeting TB preventive treatment among migrants from countries with high TB incidence moving to lower TB incidence settings. In many low-incidence countries, migrants have elevated TB incidence rates as a result of infection acquired before or during migration [10]. For example, in the United States (U.S.), 71% of reported TB cases between 2017 and 2021 occurred in non-U.S.-born persons [11], and most were attributable to infection acquired before entry [12]. Screening and treatment of *Mtb* infection in high-risk migrant populations has been identified as a key component in global tuberculosis control effort [13]. In this setting, HrTS may be useful as a rule-out test to reduce the need to treat all individuals with a positive IGRA result, the majority of whom are at very low risk of proximal progression. It could also be used as a stand-alone rule-in test for incipient TB. To the best of our knowledge, no study has evaluated the cost-effectiveness of such a test as a post-arrival screening tool for *Mtb* infection among migrants to low-TB burden countries.

In this study, we assessed the potential health impact and cost-effectiveness of four post-arrival screening strategies for *Mtb* infection among new U.S. migrants, using a potential HrTS that meets WHO TPP optimal criteria. To compare these strategies, we used a decision analytic framework with a discrete-event simulation (DES) model. We parameterized this model with data for the 2019 immigrant cohort to estimate lifetime TB-related health outcomes and health service utilization under each screening scenario.

## Methods

### Ethics statement

This analysis represented a secondary analysis of publicly available data, and no human subjects review was required.

### Study population

Our simulated study cohort (*n* = 2,042,225) included migrants whose annual TB risk could be estimated from a published TB risk model as a function of migrants’ country-of-origin, entry year, age at entry, and number of years since entry to the United States [14]. Our study cohort represented 97 countries-of-origin, accounting for 70.6% of the 2019 entry cohort and the majority of TB cases among this population. The population sizes by age and country-of-origin were estimated from 2019 American Community Survey data. We divided the study population into four risk categories based on WHO-estimated country-of-origin TB incidence rates in 2019 (I, 0–9.9; II, 10–99.9; III, 100–299.9; and IV, ≥300 per 100,000). We conducted our analyses for the entire study cohort and by risk category (Table 1). Further details are given in Appendix 1 in S1 Text.

**Table 1:** Characteristics of study cohort upon entry to the United States in 2019.

Risk category^1^	Number of countries/regions	Countries/regions^2^	Modeled population size, n (% of total population)	Mean entry age (years)
Entire cohort	97		2,042,225 (100%)	29.0
I	5	IDN, LBR, MMR, PHL, ZAF	100,778 (4.9%)	34.0
II	19	AFG, BGD, BOL, CMR, ETH, GHA, HTI, IND, KEN, KHM, LAO, NGA, NPL, PAK, PER, SLE, SOM, THA, VNM	340,454 (16.7%)	28.9
III	50	ALB, ARG, ARM, BGR, BIH, BLR, BLZ, BRA, CHL, CHN, COL, CPV, CRI, DOM, ECU, EGY, ESP, FJI, GTM, GUY, HKG, HND, IRN, IRQ, JPN, KOR, LBN, LKA, LTU, MAR, MDA, MEX, MYS, NIC, PAN, POL, PRT, ROU, RUS, SDN, SLV, SYR, TTO, TUR, TWN, UKR, URY, UZB, VEN, YEM	1,409,531 (69.0%)	27.9
IV	23	AUS, AUT, BEL, BRB, CAN, CHE, CUB, CZE, DEU, FRA, GBR, GRC, GRD, HRV, HUN, IRL, ISR, ITA, JAM, JOR, NLD, SAU, SWE	191,462 (9.4%)	34.7

^1^Epidemiological categorization of migrant populations based on TB incidence per 100k in 2019 for their country-of-origin: risk category I (≥300); risk category II (100–299.9), risk category III (10–99.9), risk category IV (0–9.9).

^2^Countries and regions are presented in ISO 3166 alpha-3 code [30]. Countries underlined are the top five country-of-origins that contribute to the total number of TB cases among migrants.

### Intervention strategies

**Fig 1:**
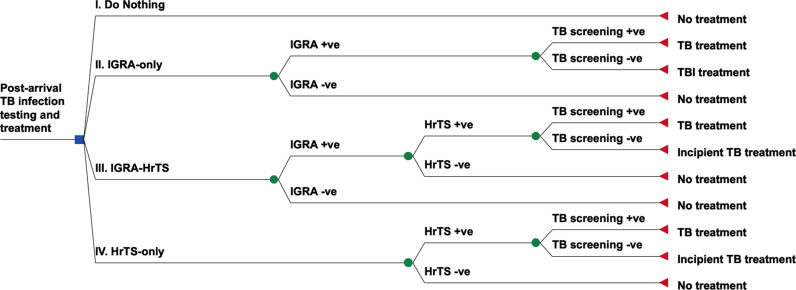
Decision tree of the four post-arrival screening strategies evaluated in our study. Abbreviations: IGRA, interferon-gamma release assays; HrTS, host-responses based transcriptional signatures; +ve, positive; −ve, negative; TBI, *Mtb* infection.

We compared four post-arrival screening strategies (Fig 1): (I) no screening for TB infection or incipient TB (‘no-screening’); (II) screen all with IGRA, provide TB preventive treatment for individuals testing positive (‘IGRA-only’); (III) screen all with IGRA followed by HrTS for IGRA-positive persons, provide incipient TB treatment for individuals testing positive with both tests (‘IGRA-HrTS’); and (IV) screen all with HrTS, provide incipient TB treatment for individuals testing positive (‘HrTS-only’). Screening was assumed to occur one month after U.S. arrival. Strategies II–IV also assumed screening and treatment for TB disease prior to the provision of TB preventive treatment or incipient TB treatment. We assumed that, if diagnosed, TB disease would be treated with a standard first-line treatment, *Mtb* infection would be treated with once-weekly isoniazid-rifapentine for 12 weeks, and incipient TB would be treated with one month of daily isoniazid-rifampicin followed by 3 months of isoniazid-rifampicin thrice weekly [15]. Table 2 summarizes clinical decision rules, and Appendix 2 in S1 Text provides a flowchart for each strategy.

**Table 2:** Testing and treatment decision rules.

Tests performed	Test results			Diagnosis, conditional on test results	Treatment prescribed, conditional on diagnosis^1^,^2^
	IGRA	HrTS	TB diagnosis		
Strategy II (IGRA → TB diagnosis)	pos	--	pos	TB disease	Treatment for TB disease
	pos	--	neg	*Mtb* infection	Treatment for *Mtb* infection
	neg	--	--	No *Mtb* infection	No treatment
Strategy III (IGRA → HrTS → TB diagnosis)	pos	pos	pos	TB disease	Treatment for TB disease
	pos	pos	neg	Incipient TB	Treatment for incipient TB
	pos	neg	--	Non-progression *Mtb* infection	No treatment
	neg	--	--	No *Mtb* infection	No treatment
Strategy IV (HrTS → TB diagnosis)	--	pos	pos	TB disease	Treatment for TB disease
	--	pos	neg	Incipient TB	Treatment for incipient TB
	--	neg	--	No incipient TB	No treatment
In all strategies, in the case of TB diagnosis that occurred outside of the post-arrival screening	--	--	pos	TB disease	Treatment for TB disease

^1^TB diagnosis is assumed to be perfect.

^2^In this study, we assumed individuals were given 3HP (once-weekly isoniazid-rifapentine for 12 weeks) for treatment for *Mtb* infection and one month of HR daily followed by three months of HR three times a week (1-month initial phase of daily isoniazid- rifampicin, followed by 3-month continuation phase of isoniazid-rifampicin) for treatment for incipient TB, a regimen that has been used for sputum negative TB cases [15].

Abbreviations: pos, positive; neg, negative; IGRA, interferon-gamma release assay; HrTS, host-response-based transcriptional signature; *Mtb*, *Mycobacterium tuberculosis*.

For IGRA, we defined sensitivity and specificity with respect to the *Mtb* infection status at the time of testing. We assumed IGRA would be positive in 89% (95%CI: 84–94) of individuals with *Mtb* infection and negative in 98% (95%CI: 95–99) of individuals without *Mtb* infection [16]. For HrTS, we defined sensitivity and specificity of HrTS with respect to prevalent and future TB disease. For specificity, we assumed that HrTS would be negative in 90% of individuals without infection or with infection that would not lead to disease before death. We assumed HrTS would have a sensitivity of 90% for prevalent TB as well as TB cases occurring within two years of testing, and a sensitivity of 10% for cases occurring subsequently. This assumption implies the test cannot distinguish individuals who will from those who will not develop TB disease beyond the two-year predictive period, consistent with the reported performance of current signatures [17]. Further, we assumed independence in IGRA and HrTS test results, conditional on an individual’s TB-related health status at the time of testing ((1) no *Mtb* infection, (2) *Mtb* infection that will not progress to TB disease during lifetime, (3) *Mtb* infection that will progress within two years (incipient TB), (4) *Mtb* infection that will progress after two years, (5) prevalent TB disease).

### Discrete-event simulation (DES) model

Each migrant in the study population was represented as an individual in the DES model, characterized by country-of-origin, entry age, and initial health state upon U.S. arrival. The three possible initial health states were: (1) no *Mtb* infection, (2) *Mtb* infection without TB disease, (3) TB disease. Initial health states were assigned based on the estimated prevalence of *Mtb* infection and estimated incidence of TB disease, by age and country-of-origin.

To parameterize the progression from *Mtb* infection to TB disease, we estimated individual TB risk over time by applying the demographic data of the 2019 entry cohort to the fitted TB risk model reported in Hill and colleagues [14]. This empirical study estimated TB incidence rates among U.S. migrants after entry to the United States, as a function of entry year, entry age, country-of-origin, and time since entry (Appendix 1 in S1 Text)*.* We assumed that the fraction of these cases attributable to *Mtb* infection present at entry to the United States (and therefore avertable by the post-arrival screening strategies) declined over time, such that for an individual present in the country for 20 years, 50% of incident TB disease would be attributed to an infection present at entry (3.4% annual decline). We also examined an optimistic scenario (all future TB incidence attributable to infections present at entry), and a pessimistic scenario (for individuals present in the country for 20 years, 10% of incident TB disease attributed to infections present at entry (10.9% annual decline)) for this assumption. Results for these scenarios are presented as sensitivity analyses.

To estimate *Mtb* infection prevalence of by country-of-origin and age group, we fit a functional relationship between *Mtb* infection prevalence and TB incidence rate using data reported by the U.S. TB Epidemiological Studies Consortium [18], and calibrated this to match the overall prevalence of *Mtb* infection in the non-US-born population [19]. See Appendix 3 in S1 Text for details. The fraction of migrants entering the United States with TB disease was based on the number of TB cases diagnosed in the six months following U.S. entry, estimated using the published TB risk model [14].

At the beginning of the simulation, each individual was assigned a time-to-TB value, drawn randomly from empirical survival functions of TB incidence (Appendix 4 in S1 Text). Each individual was also assigned a time-to-death value, drawn from survival functions derived from the 2017 U.S. Life Tables for non-U.S.-born individuals [20]. Whether a simulated individual would develop TB disease in their lifetime was determined by the Gillespie algorithm [21]: in the absence of intervention, those with a time-to-TB shorter than the time-to-death were expected to develop TB during their lifetime. TB related event data were recorded for all modeled individuals over their simulated lifetime from U.S. entry to death. TB related events included TB disease onset; testing and treatment for *Mtb* infection, incipient TB, and TB disease; and changes in TB-related health states, where applicable (Appendix 4 in S1 Text). The model was developed in R [22]. Appendix 5 in S1 Text and Table 3 report parameter values.

**Table 3:** Parameters for test performance and treatment cascade.

Parameter definitions	Point estimate (95% interval)	Source
**Test performance parameters**		
IGRA		
*P (IGRA-negative* | *no TB infection)*	0.98 (0.96–0.99)	[31]
*P (IGRA-positive* | *TB infection)*	0.89 (0.84–0.93)	[31]
Host-response-based transcriptional signature (HrTS)		
*P (HrTS-negative* | *no TB infection)*	0.90	[7]
*P (HrTS-positive* | *TB)*	0.90	[7]
*P (HrTS-positive* | *TB infection, time-to-TB <= 2 years)*	0.90	[7]
*P (HrTS-positive* | *TB infection, time-to-TB > 2 years)*	0.10	Assumption^1^
TB diagnosis		
*P (TB disease-negative* | *no TB infection)*	1.00	Assumption
*P (TB disease-negative* | *TB infection)*	1.00	Assumption
*P (TB disease-positive* | *TB)*	1.00	Assumption
**Care cascade parameters**		
*Mtb* infection treatment		
*P (initiates treatment* | *diagnosed with Mtb infection)*	0.762 (0.750–0.773)	[32]
*P (completes treatment* | *initiates treatment)*	0.903 (0.886–0.919)	[33]
*P (cured* | *completes treatment)*	0.64 (0.27–0.82)	[34]
*P (cured* | *does not complete treatment)*	0.00	Assumption
Incipient TB treatment^2^		
*P (initiates treatment* | *diagnosed with incipient TB)*	0.881 (0.760–0.905)	Assumption
*P (completes treatment* | *initiates treatment)*	0.916 (0.860–0.950)	Assumption
*P (cured* | *completes treatment)*	0.64 (0.27–0.82)	Assumption
*P (cured* | *does not complete treatment)*	0.00	Assumption
TB disease treatment		
*P (dies before diagnosis* | *incident TB)*	0.016 (0.014–0.018)	Surveillance data^3^
*P (initiates treatment* | *diagnosed with TB disease)*	1.00	Assumption
*P (dies on treatment* | *initiates treatment)*	0.054 (0.035–0.074)	Surveillance data^3^
*P (completes treatment* | *does not die on treatment)*	0.916 (0.900–0.940)	Surveillance data^3^

^1^It is assumed that the test cannot distinguish cases from non-cases of future TB disease occurring > 2 years from time of testing.

^2^Additional rationale for assumptions given in supplement Table A2 in S1 Text.

^3^Based on 2022 CDC data for non-US born population residing in the United States for less than 10 years, provided by Julie Self, Lauren Lambert, and Bob Pratt from the U.S. CDC Surveillance Team, Division of Tuberculosis Elimination, 2022-10-06.

Abbreviations: IGRA, interferon-gamma release assay; HrTS, host-response-based transcriptional signature; *Mtb, Mycobacterium tuberculosis*.

### Positive predictive value and negative predictive value

For each screening strategy, we calculated the PPV and negative predictive value (NPV) of ever having TB in their lifetime, including prevalent TB disease at the time of testing. Additionally, we estimated the PPV and NPV of having TB disease within two years of testing, including prevalent TB disease.

### Health outcomes and economic evaluation

We estimated the number of TB cases averted, quality-adjusted life years (QALYs) gained, number of each test administered, and number of each treatment prescribed under each strategy. In the base case scenario, IGRA and HrTS were $62 and $30 in 2021 U.S. dollars, respectively. Other healthcare-related and non-healthcare-related costs are in Appendix 5 in S1 Text. We conducted a cost-effectiveness analysis from both societal and healthcare sector perspectives, with costs and QALYs discounted at 3% annually [23]. We also calculated the net monetary benefit (total monetized health benefits minus total costs) for each strategy with health gains valued at $150,000 per QALY [24], and other values considered in sensitivity analyses. Appendix 6 in S1 Text provides an impact inventory and additional costing methods. This study is reported as per the Strengthening the Consolidated Health Economic Evaluation Reporting Standards 2022 (CHEERS 2022) Statement (S1 File CHEERS Checklist).

### Sensitivity analysis

We conducted probabilistic sensitivity analysis (PSA) to determine the robustness of our results to collective uncertainty in all parameter inputs, generating 1,000 simulated values for all cohort outcomes [25]. The distributions of parameters included in the PSA are in Table A3 in S1 Text. We conducted an additional two-way sensitivity analysis over the cost of HrTS ($15–$300) and willingness-to-pay threshold ($30,000–$150,000 per QALY gained). We also performed a multi-way sensitivity analysis across plausible ranges of sensitivity (75%–100%), specificity (75%–100%), and cost of HrTS ($15–$300), along with the timeframe over which the sensitivity value holds (2–10 years) and the willingness-to-pay threshold. The multi-way sensitivity analysis helps inform whether and under what circumstances the HrTS strategies would be cost-effective. Finally, we estimated results for an alternative scenario that assumed a more rapid decline in the rate of progression to TB disease with increasing years since U.S. entry. In this scenario, the annual number of TB cases remained the same as in the main analysis, but the proportion of cases attributable to a pre-existing infection was assumed to decline by 3.4% with each additional year since entry. The remainder were assumed to result from TB (re)infection after U.S. entry, and could not be averted by the modeled interventions.

Point estimates for all reported outcomes were calculated as the mean of the 1,000 values generated by the PSA, with uncertainties expressed in 95% credible intervals, unless otherwise specified. The ICER was calculated as the ratio of the mean of incremental cost to the mean of the incremental QALY gained.

## Results

### Expected number of TB cases identified by years since entry

The median age at entry of the study cohort was 29 years old, and the estimated LTBI prevalence was 12.4% (95% credible interval (CI) 8.4–43.5). Under the no-screening strategy, we projected 5,601 (3,669–8,177) members of the study cohort would develop TB over their lifetime (2.7 per 1,000, 2.4–3.1). For these individuals, the expected median time to TB was estimated to be 11.3 (interquartile range (IQR) 3.1, 26.3) years, with 20.2% (18.6–21.7) of cases occurring within two years of U.S. arrival. The CDC Online Tuberculosis Information System Data reported 840 TB cases among non-U.S.-born population within the first year they entered the United States in 2019, and our risk model estimated 770 (95% CI 629–894) cases [18].

**Fig 2:**
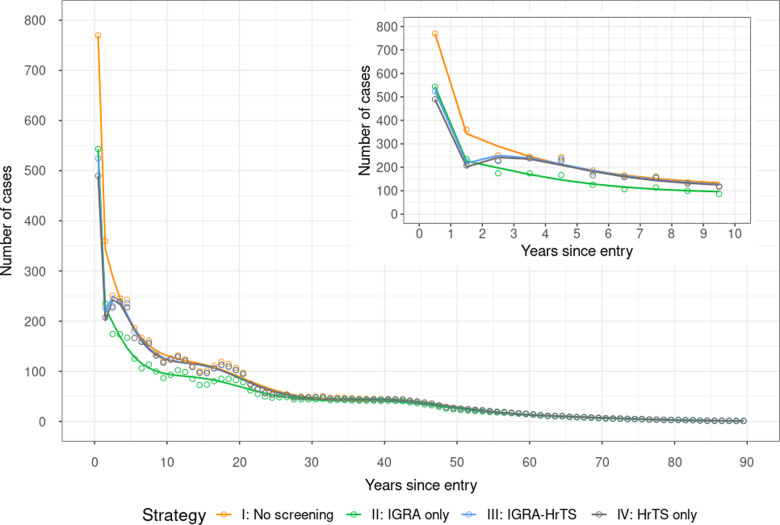
Number of TB cases, by years since entry. The circles represent the expected number of TB cases in each year, and the lines are fitted smooth lines to show the trend. IGRA, interferon-gamma release assay; HrTS, host-response-based transcriptional signature.

Fig 2 shows the projected number of TB cases by year under each strategy. In the no-screening and the IGRA-only strategies, the trend decreases monotonically. In contrast, the IGRA-HrTS and the HrTS-only strategies show a slight rebound after year one followed by a monotonic decrease thereafter, as HrTS identified most of the incipient TB at the time of screening. For the IGRA-HrTS and HrTS-only strategies, TB incidence reductions are concentrated in the years following screening, while the TB incidence reduction accumulates over a longer period, proportional to overall incidence trends, in the IGRA-only strategy.

Compared to the no-screening strategy, the IGRA-only strategy was estimated to result in the greatest reduction (23.1%, 18.7–28.8) in TB cases over the lifetime of the entire study cohort, followed by the HrTS-only strategy (10.6%, 9.4–12.2) and the IGRA-HrTS strategy (9.1%, 8.3–10.5) (Table 4). This pattern was consistent across risk categories.

**Table 4:** Testing and treatment outcomes.

	PPV for future TB disease (%, 95% CI)		NPV for future TB disease (%, 95% CI)		Percentage of population treated, by regimen type (%, 95% CI)			Reduction in TB cases (%, 95% CI)
	Within 2 years	Over lifetime	Within 2 years	Over lifetime	*Mtb* infection	Incipient TB	TB disease	
**Entire cohort**								
** *No screening***	NA	NA	NA	NA	NA	NA	0.3 (0.2, 0.3)	*ref*
** *IGRA only***	0.4 (0.3, 0.4)	1.2 (1.0, 1.4)	100 (100, 100)	100 (100,100)	9.8 (8.6, 11.5)	NA	0.2 (0.2, 0.2)	23.1 (18.7, 28.8)
** *IGRA-HrTS***	3.4 (2.8, 3.9)	4.2 (3.5, 4.9)	100 (100, 100)	99.9 (99.9, 99.9)	NA	1.2 (1.0, 1.4)	0.2 (0.2, 0.3)	9.1 (8.3, 10.5)
** *HrTS only***	0.5 (0.4, 0.5)	0.6 (0.6, 0.7)	100 (100, 100)	99.9 (99.9, 99.9)	NA	8.8 (7.8, 9.6)	0.2 (0.2, 0.3)	10.6 (9.4, 12.2)
**Risk category^1^ I**								
** *No screening***	NA	NA	NA	NA	NA	NA	0.9 (0.8, 1.0)	*ref*
** *IGRA only***	1.0 (0.8, 1.1)	3.1 (2.6, 3.5)	100 (100, 100)	99.9 (99.9, 99.9)	12.9 (11.7, 14.5)	NA	0.7 (0.6, 0.8)	22.6 (18.3, 28.3)
** *IGRA-HrTS***	8.3 (7.1, 9.6)	10.6 (9.1, 12.1)	100 (100, 100)	99.6 (99.5, 99.6)	NA	1.6 (1.3, 1.8)	0.8 (0.7, 0.9)	10.0 (8.8, 11.2)
** *HrTS only***	1.6 (1.4, 1.9)	2.1 (1.9, 2.4)	100 (100, 100)	99.6 (99.5, 99.6)	NA	9.0 (8.0, 9.8)	0.8 (0.7, 0.9)	11.6 (9.9, 12.8)
**Risk category II**								
** *No screening***	NA	NA	NA	NA	NA	NA	0.5 (0.4, 1.0)	*ref*
** *IGRA only***	0.7 (0.5, 0.8)	1.9 (1.7, 2.2)	100 (100, 100)	100.0 (99.9, 100.0)	11.9 (10.7, 13.4)	NA	0.4 (0.3, 0.8)	25.2 (20.0, 32.1)
** *IGRA-HrTS***	5.5 (4.5, 6.4)	7.1 (5.7, 8.2)	100 (100, 100)	99.8 (99.8, 99.8)	NA	1.4 (1.2, 1.7)	0.5 (0.4, 0.9)	10.0 (9.2, 10.5)
** *HrTS only***	1.0 (0.8, 1.1)	1.2 (1.0, 1.4)	100 (100, 100)	99.8 (99.7, 99.8)	NA	8.9 (7.9, 9.7)	0.5 (0.4, 0.9)	10.7 (9.5, 11.2)
**Risk category III**								
** *No screening***	NA	NA	NA	NA	NA	NA	0.2 (0.2, 1.0)	*ref*
** *IGRA only***	0.3 (0.2, 0.3)	0.9 (0.7, 1.0)	100 (100, 100)	100 (100, 100)	9.7 (8.5, 11.3)	NA	0.2 (0.1, 0.8)	22.0 (18.1, 26.9)
** *IGRA-HrTS***	2.5 (2.1, 2.8)	3.0 (2.6, 3.5)	100 (100, 100)	99.9 (99.9, 99.9)	NA	1.1 (0.9, 1.4)	0.2 (0.2, 0.9)	8.3 (7.7, 10.2)
** *HrTS only***	0.4 (0.3, 0.4)	0.4 (0.4, 0.5)	100 (100, 100)	99.9 (99.9, 99.9)	NA	8.8 (7.8, 9.5)	0.2 (0.2, 0.9)	10.3 (9.3, 12.6)
**Risk category IV**								
** *No screening***	NA	NA	NA	NA	NA	NA	0.01 (0.01, 1.01)	*ref*
** *IGRA only***	0.03 (0.02, 0.04)	0.1 (0.1, 0.2)	100 (100, 100)	100 (100, 100)	5.6 (4.4, 7.4)	NA	0.01 (0.01, 0.82)	17.8 (7.4, 33.3)
** *IGRA-HrTS***	0.3 (0.2, 0.4)	0.3 (0.2, 0.4)	100 (100, 100)	100 (100, 100)	NA	0.7 (0.5, 0.9)	0.01 (0.01, 0.92)	7.5 (2.5, 14.3)
** *HrTS only***	0.03 (0.03, 0.04)	0.03 (0.03, 0.04)	100 (100, 100)	100 (100, 100)	NA	8.6 (7.6, 9.4)	0.01 (0.01, 0.90)	7.7 (2.5, 14.3)

^1^Risk categories categorize migrant populations based on TB incidence per 100k in 2019 for their country-of-origin*:* risk category I (≥300); risk category II (100–299.9), risk category III (10–99.9), risk category IV (0–9.9).

Abbreviations: PPV, positive predictive value; NPV, negative predictive value; CI, credible interval; IGRA, interferon-gamma release assay; HrTS, host-response-based transcriptional signature; *Mtb*, *Mycobacterium tuberculosis*; NA, not applicable.

### Utilization of screening and treatment resources

In the IGRA-only strategy, 12.8% (95% CI 11.3–15.0) of the study cohort tested positive with IGRA, 1.3% (95% CI 1.2–1.5) tested positive with both IGRA and HrTS in the IGRA-HrTS strategy, and 10.01 (95% CI 9.99–10.02) tested positive with HrTS in the HrTS-only strategy (Appendix 7 in S1 Text). The proportions of the cohort receiving an intervention to prevent disease progression were more than eight times higher in the IGRA-only strategy (9.8%, 8.6–11.5) and the HrTS-only strategy (8.8%, 7.8–9.6), compared to the IGRA-HrTS strategy (1.2%, 1.0–1.4) (Table 4).

The expected proportions treated for TB disease, identified through active or passive screening, were highest in the no-screening strategy, followed by the IGRA-HrTS strategy, and the IGRA-only strategy (Table 4).

### PPV and NPV of screening algorithms

Overall, the PPV for TB disease within two years (3.4%, 2.8–3.9) and over the lifetime (4.2%, 3.5–4.9) were highest in the IGRA-HrTS strategy. The PPV for TB disease within two years was the lowest in the IGRA-only strategy (0.4%, 0.3–0.4) and the PPV for TB disease over lifetime was the lowest in the HrTS-only strategy (0.6%, 0.6–0.7). Comparing across risk groups, PPV were higher among migrant populations from countries with higher TB risks (Table 4). The NPV for future TB disease within two years and over lifetime were greater in lower risk categories, but were high overall for all strategies (Table 4).

### Health benefits, costs, and cost-effectiveness

Relative to the no-screening strategy, the IGRA-only strategy produced the greatest per-person gain in QALYs whereas the HrTS-only strategy offered the least. From both the healthcare sector and societal perspectives, the IGRA-only strategy incurred the greatest additional costs while the IGRA-HrTS strategy incurred the least. Compared to the no-screening strategy, the IGRA-only strategy resulted in an ICER of $104,138 per QALY gained in the healthcare sector perspective and an ICER of $143,103 in the societal perspective and was the optimal strategy for a willingness-to-pay of $150,000 per QALY gained. The IGRA-HrTS strategy was optimal at a willingness-to-pay of $100,000 per QALY, and the HrTS-only strategy was dominated (Table 5). When we stratified the analysis according to TB incidence rate in the country-of-origin, we found the IGRA-only strategy to be optimal for country-of-origin incidence >100 per 100,000 (i.e., cost-effective at a threshold of $150,000 per QALY gained), and the no-screening strategy optimal for country-of-origin incidence <10 per 100,000 (Appendix 8 in S1 Text). The IGRA-HrTS strategy was potentially cost-effective for migrants with country-of-origin incidence of 10–100 per 100,000, though this result had substantial uncertainty.

**Table 5:** Reference case cost-effectiveness results (time horizon: lifetime; costs and health effects incremental to the ‘no screening’ strategy, discounted at 3% annually).

Strategy	TB related HC costs^1^	Other HC expenditures^1^	TB related non-HC costs^1^	Other non-HC expenditures^1^	Productivity gain^1^,^2^	Total costs^1^,^3^ $	Total QALY gain^1^	Inc. cost^4^	Inc. effectiveness^3^ (QALYs)	ICER	Inc. NMB^5^
**Healthcare sector perspective**											
**IGRA-HrTS**	72.4 (56.6, 90.6)	0.9 (0.5, 1.4)	--	--	--	73.3 (57.6, 91.5)	0.00076 (0.00041,0.00115)	73.3	0.00076	96,447	40.0
**IGRA only**	100.6 (74.1, 131.8)	2.9 (1.5, 4.3)	--	--	--	103.5 (77.2, 134.2)	0.00105 (0.00068,0.00146)	30.2	0.00029	104,138	53.5
**HrTS only**	76.1 (56.1, 102.3)	1.0 (0.5, 1.5)	--	--	--	77.1 (57.1, 103.3)	0.00073 (0.00038,0.00115)	*NA*	*NA*	Dominated	32.8
**Societal perspective**											
**IGRA-HrTS**	72.4 (56.6, 90.6)	0.9 (0.5, 1.4)	1.3 (0.7, 1.9)	4.2 (2.1, 6.4)	4.4 (1.3,7.9)	74.4 (58.6, 92.9)	0.00076 (0.00041,0.00115)	74.4	0.00076	97,895	38.9
**IGRA only**	100.6 (74.1, 131.8)	2.9 (1.5, 4.3)	8.7 (6.4, 11.4)	11.2 (5.6, 16.3)	7.5 (3.1,10.6)	115.9 (89.0, 148.4)	0.00105 (0.00068,0.00146)	41.5	0.00029	143,103	41.1
**HrTS only**	76.1 (56.1, 102.3)	1.0 (0.5, 1.5)	12.6 (10.1, 15.4)	4.5 (2.3, 6.9)	4.7 (1.5,8.3)	89.6 (69.1, 115.7)	0.00073 (0.00038,0.00115)	*NA*	*NA*	Dominated	20.3

Abbreviations: HC, healthcare; QALY, quality-adjusted life years; ICER, incremental cost-effectiveness ratio; NMB, net monetary benefit; NA, not applicable; IGRA, interferon-gamma release assay; HrTS, host-response-based transcriptional signature.

^1^All costs, expenditures, productivity gain, and QALY gain were estimated relative to the “No Screening” strategy.

^2^Productivity gain was attributable to averted mortality.

^3^For analysis in the healthcare sector perspective, Total Costs = (TB related healthcare costs + Other healthcare expenditures); for analysis in the societal perspective, Total Costs = (TB related healthcare costs + Other healthcare expenditures + TB related non-healthcare costs + Other non-healthcare expenditures − Productivity gain).

^4^The Inc. cost and Inc. effectiveness were estimated relative to the next most costly strategy after removing dominated strategies.

^5^The NMB were calculated based on $150,000/QALY; Inc. NMB = (Inc. effectiveness * $150,000/QALY) − Inc. costs, where Inc. NMB and Inc. effectiveness here were calculated relative to the “No Screening” strategy.

### Sensitivity analysis

Two-way sensitivity analyses show that when examined by subgroups, cost-effectiveness conclusions were generally robust to the cost of HrTS as well as the willingness to pay threshold. However, when the cost of HrTS was below $15 and the willingness to pay threshold below $50,000 per QALY gained, the HrTS-only strategy was found to be the preferred strategy for the highest risk group (country-of-origin TB incidence >300 per 100,000) (Figs A2-1, A2-2 in S1 Text).

Our four-way sensitivity analyses show that for the highest risk group, at the willingness to pay threshold of $150,000 per QALY gained and HrTS costing $15, the HrTS-only strategy may be cost-effective when HrTS has a >75% sensitivity for TB cases occurring five years after testing from the societal perspective, or seven years when analyzed from the healthcare sector perspective (Figs A3-1-1, A3-1-2 in S1 Text).

Under an optimistic scenario in which all future TB cases were assumed to result from *Mtb* infections present at U.S. entry, the IGRA-only was the cost-effective strategy. Under a pessimistic scenario assuming a more rapid decline in the rate of progression to TB disease following U.S. entry, the IGRA-HrTS strategy was the cost-effective strategy from both healthcare sector and societal perspectives, at a $150,000 willingness-to-pay threshold and a $30 HrTS (Tables A11, A13 in S1 Text).

## Discussion

To our knowledge, this is the first analysis to formally evaluate the cost-effectiveness of incorporating host transcriptomic signatures in post-arrival screening algorithms among migrants in a low-incidence country. In our main analysis, we found that, at a willingness-to-pay threshold of $150,000 per QALY gained, compared to the current recommendation of using IGRA to screen for and treat *Mtb* infection, it would not be cost-effective to use HrTS either as a rule-out test among IGRA positives or as a stand-alone rule-in test for incipient TB among newly arrived migrants in the United States, even if these tests meet WHO TPP optimal criteria. At a lower willingness-to-pay of $100,000 per QALY the IGRA-HrTS strategy would be the cost-effective strategy for the entire cohort, though with substantial uncertainty. Further, in subgroup analysis the IGRA-only strategy was the optimal strategy for the two highest risk groups (country-of-origin incidence >100 per 100,000), the IGRA-HrTS strategy was optimal for country-of-origin incidence of 10–100 per 100,000, and the do-nothing strategy optimal for country-of-origin incidence below 10 per 100,000.

In our study, HrTS was assumed to have a 90% sensitivity of cases occurring in the first two years after U.S. arrival, which represented 39% of cases occurring over the lifetime of our study cohort attributable to infections present at U.S. entry. HrTS only had a 10% sensitivity for the remaining of these cases. In contrast, while IGRA had a lower sensitivity for incipient cases, these tests retained an 89% sensitivity for cases occurring after this initial two-year period, a key reason why the IGRA-only strategy dominated the other screening strategies in the primary analysis.

If HrTS could predict more cases with delayed onset, these new screening tools may be cost-effective. As we report in sensitivity analyses, the HrTS-only strategy would be preferred if HrTS retains at least a 75% sensitivity for TB cases occurring over at least the subsequent 5 years. However, it is unlikely that HrTS signatures with high sensitivity over an interval longer than two years are imminent [17].

In an alternative scenario that assumed more rapid declines in the rate of progression to TB disease following U.S. entry, the health benefits estimated for the IGRA-only strategy were proportionally lower, and HrTS was found to be potentially cost-effective. In this analysis, the IGRA-HrTS strategy became the preferred strategy from both the societal and healthcare sector perspectives for a willingness-to-pay threshold for $150,000 per QALY, indicating that the cost-effectiveness of HrTS depends on the long-term health benefits achieved with the current IGRA-only strategy. Additional studies estimating the dynamics of TB progression risk many years after migration would be valuable.

We have evaluated only one specific use case of HrTS among recent migrants to low incidence settings, and HrTS may have greater value in settings with higher TB incidence. A modeling study by Sumner and colleagues showed that targeted TB preventive therapy guided by a blood transcriptomic biomarker (RISK11) may be more effective in averting TB cases than a one-off universal treatment amongst people living with HIV but would require repeat testing [26]. The modeled cohort they evaluated had a TB incidence rate much higher than in our study. They also reported that for the signature to be cost-effective, the cost of the test would need to be one-tenth of the preventive therapy regimen, assuming annual screening. HrTS could also be used to screen for active TB disease in migrant populations. However, a cost-effectiveness analysis of HrTS for TB screening in adults with suspected TB disease in the United Kingdom showed that it would not be cost-effective compared to the status quo [27]. HrTS could potentially be cost-effective in other higher-risk populations such as healthcare workers, but further studies need to be conducted. Additionally, recent advancements in TB vaccines raise issues regarding the use of ESAT-6, a key antigen in IGRA tests, as it may reduce IGRA specificity after vaccination [28,29]. Using HrTS as a rule-out test among IGRA positives in this scenario could potentially improve the overall diagnostic accuracy. Further investigation of the cost-effectiveness of HrTS in these situations is needed.

We found that the IGRA-only strategy had the lowest PPV for incipient TB disease while the HrTS-only strategy had the lowest PPV for TB disease over the remaining lifetime. The low PPV of the HrTS-only strategy relates to the assumed 90% specificity of this test among individuals who will not develop TB in their lifetime, which could produce a substantial number of false-positive diagnoses when future TB risks in the tested population are low. If HrTS specificity were higher the PPV would also be higher, and cost-effectiveness results for the HrTS-only strategy consequently more favorable. Additionally, the results on NPV and PPV highlight that both the PPV and NPV are important metrics for quantifying the overall value provided by these predictive tests. Compared to the PPV of IGRA reported in the UK PREDICT TB study (3.0%), our study found a lower PPV of IGRA (0.4% for TB within 2 years). However, in our study population, the TB incidence rate was 38 per 100,000 person-years in year 1 and 18 per 100,000 in year 2; in the PREDICT study, incidence was 932 per 100,000 in year 1 and 115 per 100,000 in year 2. Given that the PPV will be sensitive to the incidence rate in the population of interest, this difference in TB risk could explain the difference in PPV.

Our study has several strengths. First, we used an individual-based model with granular, time-dependent TB risk estimates that are a function of each migrant’s age, entry year, country-of-origin, and years since U.S. arrival. This allowed us to estimate the lifetime outcomes of the study cohort under different scenarios and evaluate the impact of HrTS in a real-world setting that has direct clinical and policy implications. The fact that the analysis captured lifetime outcomes is particularly important because HrTS is a predictive test for future TB disease; restricting the analytic timeframe could over-estimate the comparative effect of it relative to other screening tools.

Second, our analytic framework can be used to evaluate the performance of other predictive tests with sensitivity dependent on individual patient’s time to disease in a setting where the prevalence of a disease is also time-varying. Our approach showed that an individual-based DES model has the flexibility to incorporate these two time-dependent elements. Third, our functional definitions of a ‘positive test’, a ‘negative test’, and a ‘TB case’ in PPV and NPV calculations provided a clear performance measure for a predictive test for future TB disease. This can be useful in the evaluation of novel screening and diagnostic tools as the field has an increasing interest in tools that can identify individuals who are progressing from *Mtb* infection to TB disease.

One limitation of our study is that we assumed conditional independence of IGRA and HrTS test results. If this assumption does not hold, we may over-estimate the sensitivity of the IGRA-HrTS strategy. We estimated country-specific LTBI prevalence among migrants based on a previously published study that focused on high-risk populations, which might not be representative of the general migrant population. To address this limitation, we calibrated the estimates so that the LTBI prevalence of the entire migrant population in our study matched the national non-U.S.-born LTBI prevalence estimate reported in NHANES. Other key assumptions we made in our study included the treatment uptake and completion rates and regimen efficacy for incipient TB. Screening and treatment of incipient TB is not routine clinical practice, and there is little empirical evidence to inform these estimates.

Many signature tests have been proposed in recent years, with the goal of identifying individuals with a high proximal risk of developing TB disease [3,17]. Currently, none has met WHO TPP optimal criteria. Our findings suggest that post-arrival screening would not be cost-effective among migrants from countries with TB incidence <10 per 100,000 while screening with the conventional IGRA-only approach would be the optimal strategy for migrants from countries with incidence above 100 per 100,000. For migrants from countries with intermediate incidence levels (between 10 and 100 per 100,000), we found that a screening strategy incorporating HrTS could potentially be cost-effective, but this conclusion had substantial uncertainty. Research to resolve these uncertainties would be valuable to confirm the potential value of HrTS as a component of migrant screening programs.

## Supporting information

S1 Text Online Data Supplement. (DOCX)

S1 File CHEERS Checklist. (DOCX)

## Abbreviations

**:**
CHEERS 2022: Consolidated Health Economic Evaluation Reporting Standards 2022
DES: discrete-event simulation
HrTS: host-response-based transcriptional signature
ICER: incremental cost-effectiveness ratio
IGRA: interferon-gamma release assay
*Mtb*: *Mycobacterium tuberculosis*
NPV: negative predictive value
PPV: positive predictive value
QALY: quality-adjusted life-year
TB: tuberculosis
TPP: Target Product Profile
